# Modeling bile duct ischemia and reoxygenation injury in human cholangiocyte organoids for screening of novel cholangio-protective agents

**DOI:** 10.1016/j.ebiom.2022.104431

**Published:** 2023-01-04

**Authors:** Shaojun Shi, Henk P. Roest, Thierry P.P. van den Bosch, Marcel J.C. Bijvelds, Markus U. Boehnert, Jeroen de Jonge, Sven O. Dekker, Antoine A.F. de Vries, Hugo R. de Jonge, Monique M.A. Verstegen, Luc J.W. van der Laan

**Affiliations:** a Department of Surgery, Erasmus MC Transplant Institute, University Medical Center, Rotterdam 3015CE, the Netherlands; b Department of Organ Transplantation, Guangdong Provincial People's Hospital (Guangdong Academy of Medical Sciences), Southern Medical University, Guangzhou 510080, China; c Department of Pathology, Erasmus MC-University Medical Center, Rotterdam 3015CE, the Netherlands; d Department of Gastroenterology and Hepatology, Erasmus MC-University Medical Center, Rotterdam 3015CE, the Netherlands; e Department of Cardiology, Leiden University Medical Center, Leiden 2300 RA, the Netherlands

**Keywords:** Ischemia-reperfusion injury, Biliary injury, Liver transplantation, Organoid, Drug screening

## Abstract

**Background:**

Ischemia of the bile duct is a common feature in liver disease and transplantation, which represents a major cause of morbidity and mortality, especially after liver transplantation. Detailed knowledge of its pathogenesis remains incomplete due to the lack of appropriate *in vitro* models.

**Methods:**

To recapitulate biliary damage induced by ischemia and reperfusion *in vitro*, human intrahepatic cholangiocyte organoids (ICOs) were grown at low oxygen levels of 1% up to 72 h, followed by re-oxygenation at normal levels.

**Findings:**

ICOs stressed by ischemia and subsequent re-oxygenation represented the dynamic change in biliary cell proliferation, upregulation of epithelial–mesenchymal transition (EMT)-associated markers, and the evocation of phase-dependent cell death programs similar to what is described in patients. Clinical-grade alpha-1 antitrypsin was identified as a potent inhibitor of both ischemia-induced apoptosis and necroptosis.

**Interpretation:**

These findings demonstrate that ICOs recapitulate ischemic cholangiopathy *in vitro* and enable drug assessment studies for the discovery of new therapeutics for ischemic cholangiopathies.

**Funding:**

Dutch Digestive FoundationMLDS D16-26; TKI-LSH (Topconsortium Kennis en Innovatie-Life Sciences & Health) grant RELOAD, EMC-LSH19002; Medical Delta program “Regenerative Medicine 4D”; 10.13039/501100004543China Scholarship Council No. 201706230252.

**Research in context**

**Evidence before this study**

Ischemic cholangiopathy is one of the major causes of graft failure after liver transplantation. Biliary epithelial cells are believed to be highly vulnerable to ischemia and reperfusion injury. Based on multiple clinical and histological studies, biliary damage and impairment of biliary regeneration are believed to be the key events in the pathogenesis of ischemic cholangiopathy. However, the response of biliary epithelium to ischemia and reperfusion is widely believed to be dynamic and phase-dependent. Currently, the detailed molecular mechanism of ischemic injury in cholangiocytes remains largely unexplored due to the lack of an appropriate cellular or animal model.

**Added value of this study**

Human liver-derived intrahepatic cholangiocyte organoids (ICOs) represent an experimental tool for cholangiopathies modeling *in vitro*. ICOs are capable of self-organization and retain most biliary characteristics. In the present study, we reported the use of ICOs culture to model ischemic biliary injury *in vitro*. ICOs under ischemic culture, followed by re-oxygenation, showed a dynamic change in biliary cell proliferation and upregulation of epithelial–mesenchymal transition (EMT)-related markers, which is similar to that in patients’ biopsies. Distinct and phase-dependent cell death modalities, including apoptosis and necroptosis, were also observed in the ischemia and re-oxygenation phases. Based on this cellular model, we found that the caspase inhibitor failed to alleviate ischemic biliary damage but aggravate cell death by promoting necroptosis. Furthermore, clinical-grade alpha-1 antitrypsin (A1AT) was identified as a cholangio-protective agent by suppressing both apoptosis and necroptosis.

**Implications of all the available evidence**

In this study, we demonstrate that human ICOs cultures are feasible to model bile duct ischemia and reoxygenation injury *in vitro*. Our findings provide a useful tool for pathogenesis investigation and drug screening for the study of cholangiopathies.

## Introduction

Ischemia and reperfusion injury (IRI) is a detrimental process caused by insufficient blood supply, which happens mostly in liver surgery and liver transplantation. IRI could cause a focal or extensive injury to the biliary epithelium and lead to subsequent bile duct stricture, which is widely known as ischemic cholangiopathy.^1^ In the setting of liver transplantation, IRI represents a major cause of post-transplantation morbidity and mortality, with a relatively higher incidence in recipients accepting donation after cardiac death grafts, compared to donation after brain death.^2^ The ischemic biliary lesions may occur when the blood supply of small hepatic arteries or the peribiliary vascular plexus is interrupted. Biliary injury caused by IRI may manifest as biliary necrosis, bile leakage, biloma, biliary fibrosis, or stenosis.^3^ Liver transplantation remains the only curative treatment for patients with severe ischemic cholangiopathy.^4^

Cholangiocytes are believed to be more vulnerable to IRI compared to other liver parenchymal cells.^5^ It is hypothesized that not IRI itself but also bile salt toxicity and immune response invoked by IRI, are the major mechanisms of ischemic biliary injury.^6^ As a consequence of damage, massive cholangiocyte death could be induced, resulting in intercellular junction loss, detachment from the basement membrane, and even bile leakage into the liver parenchyma and bile duct strictures.^7^ The pathophysiological change of cholangiocytes caused by hepatic IRI appears to be phase-dependent. For instance, Liu et al.^8^ reported that the proliferation of biliary epithelial cells could be greatly suppressed during cold ischemia but be partially restored during reperfusion, which is consistent with the findings *in vitro*.^9^ BAX-mediated intrinsic apoptosis could be invoked by hypoxia predominantly in cholangiocytes *in vitro*,^9^ while reoxygenation promotes tumor necrosis factor-related apoptosis-inducing ligand-mediated extrinsic apoptosis instead.^10^ Biliary necrosis is also present in ischemic cholangiopathy, especially in parallel with the development of hepatic artery thrombosis, but the detailed molecular mechanism is unknown.^6^

The impairment of biliary regenerative capacity also represents a key mechanism in the pathogenesis of ischemic cholangiopathy. In fact, recent studies demonstrate that major epithelial cell loss occurs in more than 80% of donations after brain death or liver grafts at the time of transplantation, but the ischemic biliary injury is only seen in a minority of recipients (less than 20%),^11^^,^^12^ suggesting that both initial ischemic biliary injury and subsequent biliary regeneration determine the consequence of ischemic injury. Hepatic and biliary tree stem/progenitor cells, residing in bile ductules and peribiliary glands (PGB) are known to repopulate the injured biliary epithelium by proliferation and differentiation into mature cholangiocytes. Although the response of hepatic and biliary stem/progenitor cells to IRI remains unexplored, previous histological studies, based on clinical biopsies, revealed the strong association between the ischemia-induced PBG injury and post-transplant non-anastomotic strictures.^7^^,^^13^ Interestingly, profibrogenic factors present in PBG, such as transforming growth factor-beta (TGFβ), could be triggered by hypoxic damage.^13^ Moreover, epithelial-mesenchymal transition (EMT) of biliary epithelial cells was found *in vitro* and *in vivo* models after inducing ischemia, indicating the potential involvement of EMT in ischemic cholangiopathy.^14^

Clinical studies have extended our understanding of the pathogenesis of hepatic IRI in the last decades, which mainly focused on hepatocytes. However, the detailed molecular mechanism of ischemic injury in cholangiocytes remains largely unexplored due to the lack of an appropriate cellular model, hampering the development of therapeutic strategies. Liver-derived cholangiocyte organoids represent a model for human biliary epithelial cells, which are capable of self-organization and retain most biliary characteristics *in vitro*.^15^, ^16^, ^17^ We have previously described that cholangiocyte organoids can be used to recapitulate cholangiopathy-associated programmed cell death, which is induced by multiple stimuli such as tumor necrosis factor-alpha, bile acid, and ethanol metabolites.^15^ In the present study, we used ICOs to model IRI by exposing them to hypoxia and reoxygenation (H/R) insult *in vitro*. We aimed to investigate the transcriptional profiles of ICOs stressed by H/R. With an eye on programmed cell death including apoptosis and necroptosis, we further examined the biliary damage induced by H/R in ICOs and utilized this model for drug evaluation.

## Methods

### Patients, specimens, and materials

For initiation of organoid cultures, fresh donor liver biopsies (0.5∼1 cm^3^, n = 5) were collected at the time of transplantation and stored in cold University of Wisconsin solution. For immunostaining, liver specimens were obtained during transplantation at 60 min after portal reperfusion (n = 4). The use of human specimens was approved by the Erasmus MC medical ethics council (MEC-2014-060). All patients gave informed consent for the use of material for research purposes. Donors’ demographic and clinical characteristics are summarized in Table S1.

### Human cholangiocyte organoid initiation and culture

As previously described,^15^^,^^16^ ICOs (n = 5) were initiated and cultured using an established protocol. In brief, donor liver tissue was minced, rinsed twice with Earle's balanced salt solution (EBSS, Thermo-Fisher, Waltham, USA), and digested in collagenase solution (2.5 mg/mL collagenase Type A, Sigma–Aldrich, St. Louis, USA) in Advanced DMEM/F-12 medium (Invitrogen, Carlsbad, USA) for 15 min at 37 °C. Cold Advanced DMEM/F-12 was added to stop the digestion. The single-cell suspension was filtered using a 70 μm Nylon cell strainer and centrifuged at 1500 rpm, 5 min, at 4 °C. After that, the cell pellets were collected at the bottom and subsequently mixed with BME (Basement Membrane Extract, Type 2, R&D Systems, Minneapolis, USA). Cell droplets were then seeded in multi-well plates. Initiating medium was added after the solidification of BME and the cells were cultured at 37 °C in a humidified atmosphere with 5% CO^2^. After three days of culture in the initiating medium, the cells were refreshed with an expansion medium in which noggin, Wnt, Y27632, and hES cell cloning recovery supplement were deprived. Components supplemented in Advanced DMEM/F12 medium for initiating and expansion medium were listed in Table S2.

### Hypoxia and reoxygenation stimulation in ICOs

After culturing over three passages, the ICOs were transferred to a hypoxia workstation (Invivo2, Baker Ruskin, Maine, USA) to mimic the ischemia-reperfusion injury *in vitro*. ICOs were cultured in an expansion medium and exposed to hypoxia (∼1% O^2^, 5% CO^2^). The duration of hypoxia was tested ranging from 6 to 120 h (data not shown). The optimal duration of hypoxia was set at 72 h based on the response of ICOs transduced with the hypoxia-inducible factor 1 αlpha (HIF-1α) reporter. ICOs were refreshed with expansion medium and switched to normoxia condition (reoxygenation phase, 21% O^2^, 5% CO^2^) and cultured for 6 and 24 h. Candidate cell death inhibitors, including emricasan (20 μM, Selleckchem, Texas, USA) and clinical-grade A1AT (20 mg/mL, CSL Behring, Pennsylvania, USA), were co-incubated with ICOs throughout the hypoxia and reoxygenation culture. The bight-field images of ICOs were captured using the EVOS cell imaging system (Thermo-Fisher, Waltham, USA). The average diameter of stimulated organoids was determined in bight-filed images (at least 15 organoids per subgroup) in triplicate using Image J software (NIH) as described before.^15^^,^^18^

### Cell viability assay

To determine the viability of stimulated ICOs, the CellTiter-Glo 3D Cell Viability Assay (Promega, Madison, USA) was applied according to the manufacturer's instructions. Briefly, the ICOs were trypsinized into single cells and seeded in a concentration of 1.0 × 10^6^ cells/ml. After stimulation with H/R, the medium was removed and a volume of CellTiter-Glo reagent equal to the volume of the cell culture medium was added. The ICOs were disrupted vigorously for 5 min and then incubated at room temperature for 25 min avoiding the light. The luminescence were determined by a microplate reader (BMG Labtech, Durham, USA).

### Construct design, lentiviral production, and transduction

The self-inactivating lentiviral vector (LV) shuttle plasmid pLV.5×HRE.GpNLuc.WHVoPRE was generated in a multistep procedure using pRetroX-Tight-MCS_PGK-GpNLuc (Addgene, Watertown, MA; plasmid number 70185) and pLV.5×HRE.eGFP.WHVoPRE as starting constructs. pLV.5×HRE.eGFP.WHVoPRE contains 5 copies of the hypoxia-responsive element (HREs) followed by a minimal simian virus 40 promotor driving the expression of transcripts encoding the *Aequorea victoria* enhanced green fluorescent protein (eGFP). pLV.5×HRE.GpNLuc.WHVoPRE is identical to pLV.5×HRE.eGFP.WHVoPRE expects that in codes for a dual reporter protein consisting of eGFP fused at its C terminus to NanoLuc luciferase^19^^,^^20^ under hypoxic conditions. pLV.5×HRE.GpNLuc.WHVoPRE further contains an optimized version of the woodchuck hepatitis virus post-transcriptional regulatory element^21^ immediately downstream of the GpNLuc-coding sequence to enhance transgene expression. Recombinant plasmid construction was done with enzymes from New England Biolabs (Bioké, Leiden, the Netherlands) or Fermentas (Thermo Fisher Scientific, Breda, the Netherlands) using standard procedures or following the instructions provided with specific reagents. Large-scale plasmid DNA extractions were done using LabNed Plasmid Maxiprep Kits (LabNed, Amstelveen, the Netherlands) according to the manufacturer's instructions.

The generation of vesicular stomatitis virus G protein-pseudotyped self-inactivating LV particles was performed by transfecting subconfluent monolayers of HEK293T cells with one of the LV shuttle plasmids and the packaging plasmids psPAX2 (Addgene, plasmid number: 12260) and pLP/VSVG (Thermo Fisher Scientific) at a molar ratio of 2:1:1. Culturing of HEK293T cells was done in high-glucose Dulbecco's modified Eagle's medium (DMEM; Thermo Fisher Scientific; cat# 41966) supplemented with 10% fetal bovine serum (FBS; Biowest, VWR International, Amsterdam, the Netherlands; cat# S1860-500). The transfection mixture consisted of 35 μg total plasmid DNA and 105 μg PEI MAX (Polysciences Europe, Eppelheim, Germany) dissolved in 2 mL of phosphate-buffered saline (PBS) per 175-cm^2^ cell culture flask (Greiner Bio-One, Alphen aan den Rijn, the Netherlands) and was directly added to the culture medium. After 4 h, the transfection medium was replaced by high-glucose DMEM supplemented with 5% FBS and 25 mM HEPES-NaOH (pH 7.4). Harvesting of the viral particles was performed ∼48 h after initiating the transfection by collecting the HEK293T supernatants, clearing it from cellular debris by centrifugation for 10 min at 3750×*g*, and subsequent filtration through 0.45-μm pore-sized, polyethersulfone Millex-HP syringe filters (Millipore, Amsterdam, the Netherlands). To further purify and concentrate the viral particles, the supernatant was transferred to a 38.5-mL polypropylene ultracentrifuge tube (Beckman Coulter Nederland, Woerden, the Netherlands; cat# 326823), and underlay with 5 mL of a 20% (w/v) sucrose in PBS solution. These tubes were centrifuged with slow acceleration and without braking at 15,000 revolutions per minute in an SW32 rotor (Beckman Coulter Nederland) for 120 min at 4 °C. The concentrated vector suspension was divided on ice into 100 μl aliquots and stored at −80 °C until use.

For transduction, the ICOs were harvested at 70–80% confluency, disrupted mechanically, and digested with trypsin–EDTA (Gibco) into single cells at 37 °C for 5 min. The transduction was initiated by adding viral supernatant to the ICOs in 48-well plates and sealing the plate with parafilm. Spinoculation was performed by centrifuging the plate at 600×*g*, for 60 min, at room temperature. The parafilm wrapping was discarded and the plates were incubated at 37 °C for 6 h. After that, the cells were collected and spun at 900×*g*, 5 min, at 4 °C. The medium was subsequently removed, and the cells were resuspended in BME, seeded, and cultured in the expansion medium as mentioned above. For the selection of transduced cells, after the organoids grew out, ICOs were harvested and trypsinized as described before and the flow sorting was performed subsequently to select the transduced cells.

### Bulk RNA-sequencing

RNA from ICOs was collected immediately after hypoxia or reoxygenation stimulation. After removal of the medium, the ICOs in BME were washed with warm PBS three times. After that, lysis buffer (QIAzol lysing reagent, Qiagen, Hilden, Germany) was added and the RNA was isolated using the miRNeasy mini kit (Qiagen) following the manufacturer's protocol. Samples were sequenced using the Illumina Hi-Seq 4000 platform (Novogene, Beijing, China) resulting in 20–30 million, 150 nucleotides long, paired-end reads. Sequencing reads were quality controlled using FastQC and checked for P5 and P7 adapter containing sequences, and low-quality reads (N > 10% i = or Base Quality < 5). The resulting datasets were uploaded to the Galaxy Web platform public server usegalaxy.eu^22^ and mapped using HISAT2 (version 2.2.1) against the human reference genome GRCh38. Mapped reads were translated to counts using FeatureCounts (version 2.0.1) applying the built-in hg38 genome annotation file. Differentially expressed genes were identified using DESeq2 for the following pairwise factors: ‘Hypoxia’, ‘Reoxygenation’, and ‘Untreated’. Genes with an adjusted *p*-value <0.05 (Benjamini Hochberg FDR corrected) were considered differentially expressed for each individual comparison. Gene set enrichment analysis (GSEA) was performed using fgsea tool (version 1.8.0) in Galaxy. In case standardized data were required, the row z-score was calculated using the formula z = (x-μ)/σ, where x is the raw score, μ is the population mean, and σ is the population standard deviation. Raw data are available from the GEO repository at NCBI (accession number GSE198860).

### Immunostaining and imaging

#### Organoids

For whole-mount immunostaining, ICOs were washed with warm phosphate-buffered saline (PBS) and fixed in 4% PFA for 15 min, and stored in 1% PFA-PBS at 4 °C. Briefly, incubation with 1% bovine serum albumin (BSA) and 10% normal goat serum (both from Sigma–Aldrich) in PBS were performed to prevent a-specific staining. Primary antibodies were incubated with ICOs at 4 °C overnight. Secondary goat anti-rabbit 555 antibodies (Invitrogen) were subsequently diluted in a 1:1000 ratio and incubated with organoids for 1 h at room temperature. ICOs were mounted using an antifade mounting medium with DAPI (Vector Labs, Burlingame, USA). Staining of F-actin was performed using Alexa Fluor™ Phalloidin (Invitrogen) following the manufacturer's instructions. Images were captured on a confocal microscope (LSM 700, Carl Zeiss, Jena, Germany) and further processed using ZEN software (Carl Zeiss). The laser intensity was kept the same for all samples. Fluorescent images were projected with maximum intensity and at least 15 areas with cells in each sample were randomly selected. The positivity of fluorescent targets was measured using FIJI (Image J 1.51c, National Institutes of Health, Maryland, USA).

#### Paraffin-embedded liver tissue

Liver tissue biopsies (n = 4) were collected during liver transplantation and fixed in 4% PFA for 24 h, followed by dehydration, paraffin embedding, and sectioning according to standard procedures. Automated multiplex immunofluorescent staining was further performed using the Ventana Benchmark Discovery (Ventana Medical Systems Inc.). For the novel series, following deparaffinization and heat-induced antigen retrieval with CC1 (#950-500, Ventana) for 64 min at 97 °C, the tissue samples were incubated with primary antibodies at 37 °C in a step-by-step manner. The antibody denature step was performed between every antibody incubation and visualization using CC2 (#950-123, Ventana) for 20 min at 100 °C. Keratin 19 (KRT19) was incubated for 32 min, detected with Universal HQ kit (#760-275, Ventana), and visualized with Red610 (#760-245, Ventana) for 4 min. TGF-β was incubated for 32 min, detected with Universal HQ kit, and visualized with R6G (#760-244, Ventana) for 4 min. N-cadherin was incubated for 32 min, detected with omnimap anti-mouse HRP, and visualized with FAM for 4 min. Slides were incubated in PBS with DAPI for 15 min and covered with an anti-fading medium (DAKO, S3023). Slides were scanned using the ZEISS Axio Imager 2.0.

Information on the antibodies used is listed in Table S3.

### Statistical analysis

All statistical analyses were performed using Prism software (GraphPad Software Inc.). Data are presented as the mean ± standard deviation (SD) from at least three replicates. Shapiro–Wilk test was performed to examine the normality of the distribution of all data sets. A comparison between the two groups was conducted using the unpaired Student's t-test or Mann–Whitney test. Comparisons between multiple groups were performed using a one-way ANOVA test followed by Tukey's post hoc test or Kruskal–Wallis test followed by Dunn's post hoc test. For each test, *p* < 0.05 was considered statistically significant.

The figures were created using BioRender.com and Prism software.

### Role of funding source

The study was supported by grants from Dutch Digestive Foundation MLDS D16-26, TKI-LSH (Topconsortium Kennis en Innovatie-Life Sciences & Health) grant RELOAD, EMC-LSH19002, Medical Delta program “Regenerative Medicine 4D” and China Scholarship Council No.201706230252. The funders provided financial support but did not participate in the study's design, data collection, data analysis, interpretation, or article writing. We have not been paid to write this article by a pharmaceutical company or other agency.

## Results

### Development of H/R model in ICOs

**Fig. 1:**
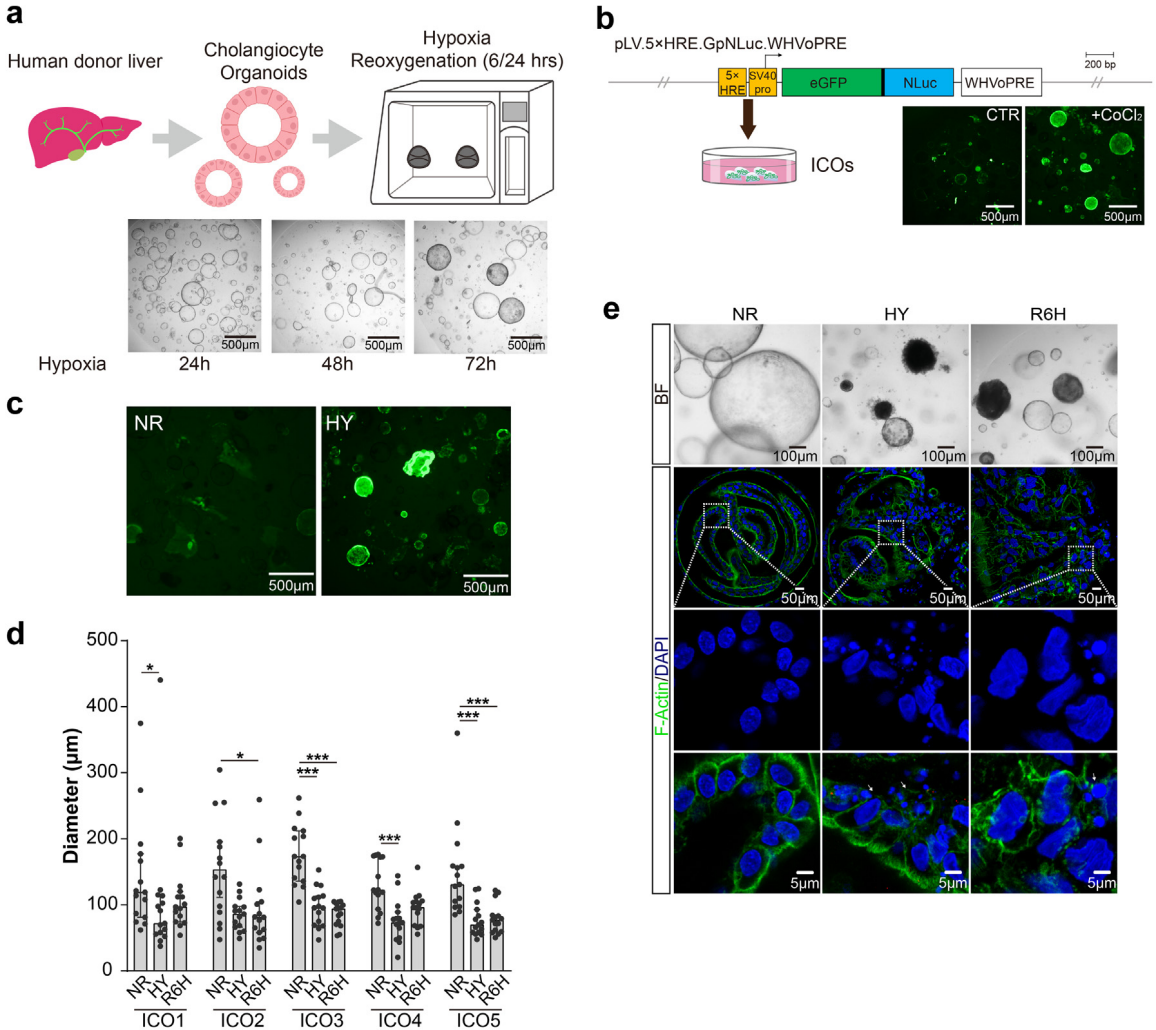
**Establishment of H/R model using human cholangiocyte organoids.** (a) Scheme of the isolation and culture of ICOs from human donor liver specimens and the procedure to establish the H/R model *in vitro*. (b) Structure of the hypoxia-responsive reporter constructs encoding a constitutively active version of human HIF-1α. ICOs were transduced with lentiviral constructs. Transduced ICOs were incubated with or without CoCl_2_ (300 μM) for 7 days. Representative fluorescent images of stimulated ICOs were shown (magnification 40×). (c) Transduced ICOs were stimulated with 72-h hypoxia. Representative fluorescent images were shown (magnification 40×). (d) ICOs (n = 5) were stimulated with 72-h hypoxia, followed by 6-h reoxygenation. The diameter of stimulated ICOs was determined in a time-course manner and analyzed using Image J software. (e) ICOs were treated for 72-h hypoxia and 6-h reoxygenation and then fixed and stained with DAPI/Phalloidin. Bright-filed and confocal imaging was shown. Enlarged images from the boxed area are shown in the bottom panel. Dying cells are indicated by arrows. Data are mean ± SD, ∗*p* < 0.05, ∗∗∗*p* < 0.001, Kruskal–Wallis test followed by Dunn's post hoc test (d). 5×HRE, 5 copies of the hypoxia-responsive element; eGFP, *Aequorea victoria* enhanced green fluorescent protein-coding sequence; HY, hypoxia; NLuc, NanoLuc-coding sequence; NR, normoxia; R6h, reoxygenation for 6 h; SV40 pro, minimal simian virus 40 promoters; WHVoPRE, optimized version of the woodchuck hepatitis virus post-transcriptional regulatory element.

To mimic IRI in organoid cultures, we used a gas-controlled hypoxia workstation in which ICOs were exposed to low oxygen tension (∼1%, HY), followed by normoxia conditions (21% O^2^, RE). Different durations of hypoxia treatment (24/48/72 h) were tested in which hypoxia for 72 h led to an obvious morphological change in ICOs (Fig. 1a). HIF-1α is the major transcription factor responding to low oxygen tension in which the HIF-1α stands out as a master regulating multiple pathways. HIF-1α expression was continuously induced by lack of oxygen but rapidly decreased in normoxia.^23^ To indicate whether hypoxia was reached in our system, ICOs were stably transduced with long-life HIF-1α-eGFP reporter constructs, which enables endogenous HIF to bind to five tandem repeats of HREs. Cobalt chloride (CoCl_2_), a hypoxia mimetic stabilizing HIF-1α,^24^ was co-incubated with transduced ICOs as a positive control, and subsequently resulted in extensive eGFP signal, suggesting successful transduction (Fig. 1b). After 72 h of incubation in the hypoxia workstation, we observed substantial expression of HIF-1α indicated by a green fluorescence signal in ICOs, revealing a clear hypoxia response (Fig. 1c). ICOs from different donors responded to H/R similarly, morphologically featured by significantly decreased organoid size (Fig. 1d), turning dark and disrupted epithelial structure (Fig. 1e). Fluorescent microscopy analysis further showed clear disintegration of the actin cytoskeleton and massive fragmentation of the nucleus in the ICOs stressed by H/R, indicating an apoptosis-like phenotype.^15^

### Hypoxia reduces cell proliferation in ICOs and this is recovered after re-oxygenation

**Fig. 2:**
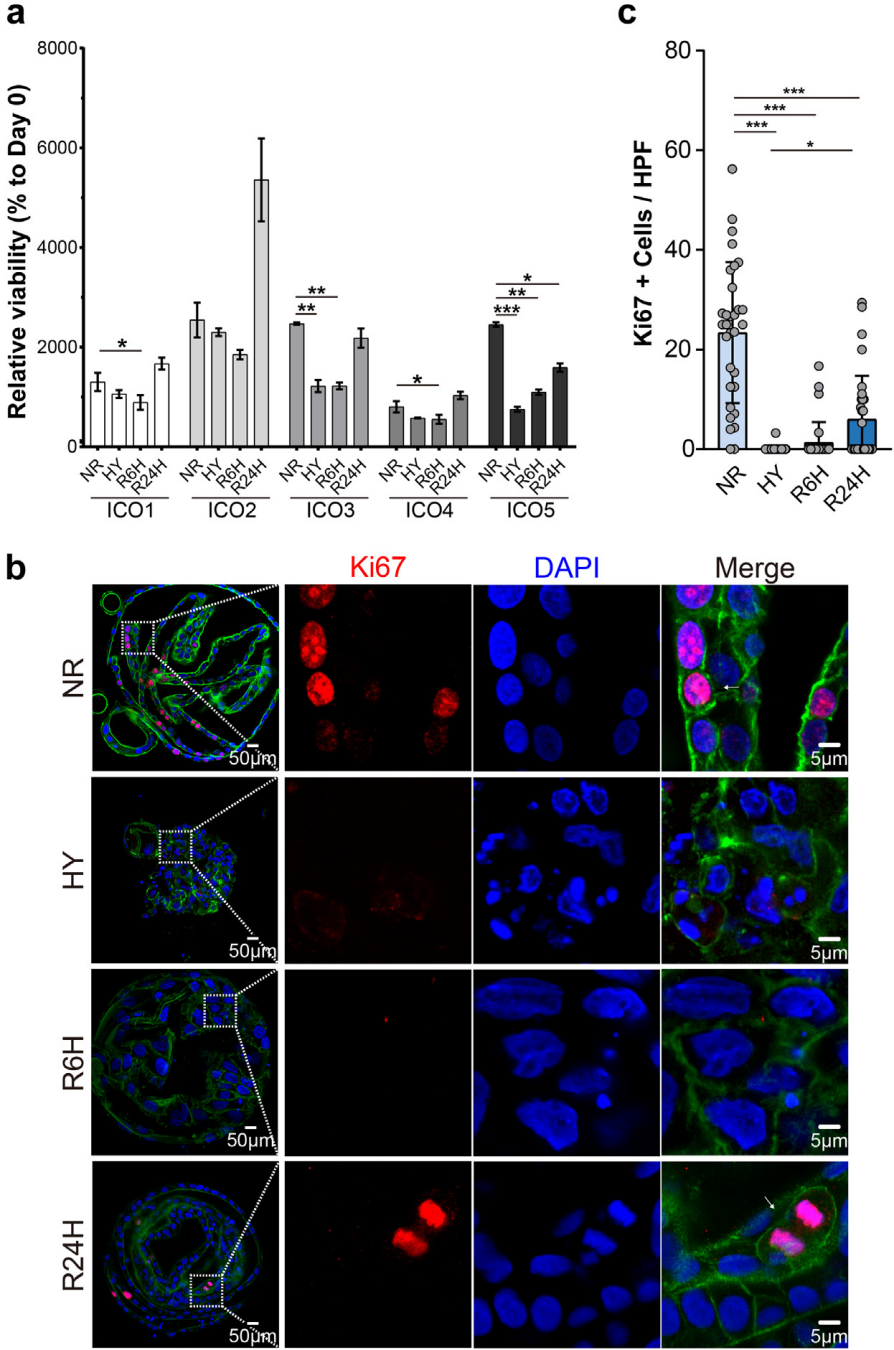
**Influence of H/R stress on ICOs proliferation.** (a) ICOs (n = 5) were stimulated with 72-h hypoxia, followed by 6/24-h reoxygenation. Cell viability measurement was performed by assessing the ATP content in the culture using the CellTiter-Glo assay. Relative cell viability was calculated by normalizing untreated ICOs at Day 0. (b) Stimulated ICOs were fixed and stained with Ki67 (red), DAPI (blue), and F-actin (green). Representative fluorescent images are shown. Enlarged images from the boxed area are shown on the right panels. (c) Quantification of Ki67 positive cells in fluorescent images was performed using Image J (at least 30 high power fields in each condition). Data are mean ± SD, ∗*p* < 0.05, ∗∗∗*p* < 0.001, Kruskal–Wallis test followed by Dunn's post hoc test (c). HPF, high power fields; HY, hypoxia; NR, normoxia; R6h, reoxygenation for 6 h; R24h, reoxygenation for 24 h.

Human ICOs resemble mature primary cholangiocytes *in vitro* but also represent cholangiocytes with higher proliferating activity and stemness due to the canonical Wnt-stimulating culture condition.^25^^,^^26^ Given that the organoid size decreased significantly after hypoxia, we further examined the change in cell viability in ICOs. Compared to ICOs in normoxia, a general reduction of cell viability in ICOs upon hypoxia stress was observed, though at different levels, depending on the donors (Fig. 2a). Interestingly, the cell viability of ICOs was increased after reoxygenation for 24 h, suggesting considerable recovery from the hypoxia-induced injury. We reasoned that hypoxic insult might reduce the usually high proliferation rate of ICOs. To prove this, we performed immunostaining of Ki67, a marker for cell proliferation (Fig. 2b). There were over 20% of ICOs expressing nuclear Ki67 under normoxic culture conditions (Fig. 2c). The number of Ki67^+^ cells dropped significantly in hypoxia (0.10 ± 0.57%, *p* < 0.001, Kruskal–Wallis test followed by Dunn's post hoc test) and 6-h reoxygenation (1.36 ± 4.10%, *p* < 0.001, Kruskal–Wallis test followed by Dunn's post hoc test) conditions, suggesting that hypoxia greatly suppressed the ICOs proliferation which was not restored by short-time reoxygenation. Interestingly, after reintroducing oxygen to ICOs for 24 h, a significant increase in the Ki67^+^ population was observed compared to ICOs under hypoxia culture (6.05 ± 8.68% vs. 0.10 ± 0.57%, *p* < 0.05, Kruskal–Wallis test followed by Dunn's post hoc test), indicating that the reduction of proliferation caused by hypoxia could be restored by the switch to normoxia. These results are consistent with a previous study in which ischemia was found to suppress biliary proliferation while reperfusion could restore it to a certain extent.^8^

### Transcriptomic analysis of ICOs upon H/R stress

**Fig. 3:**
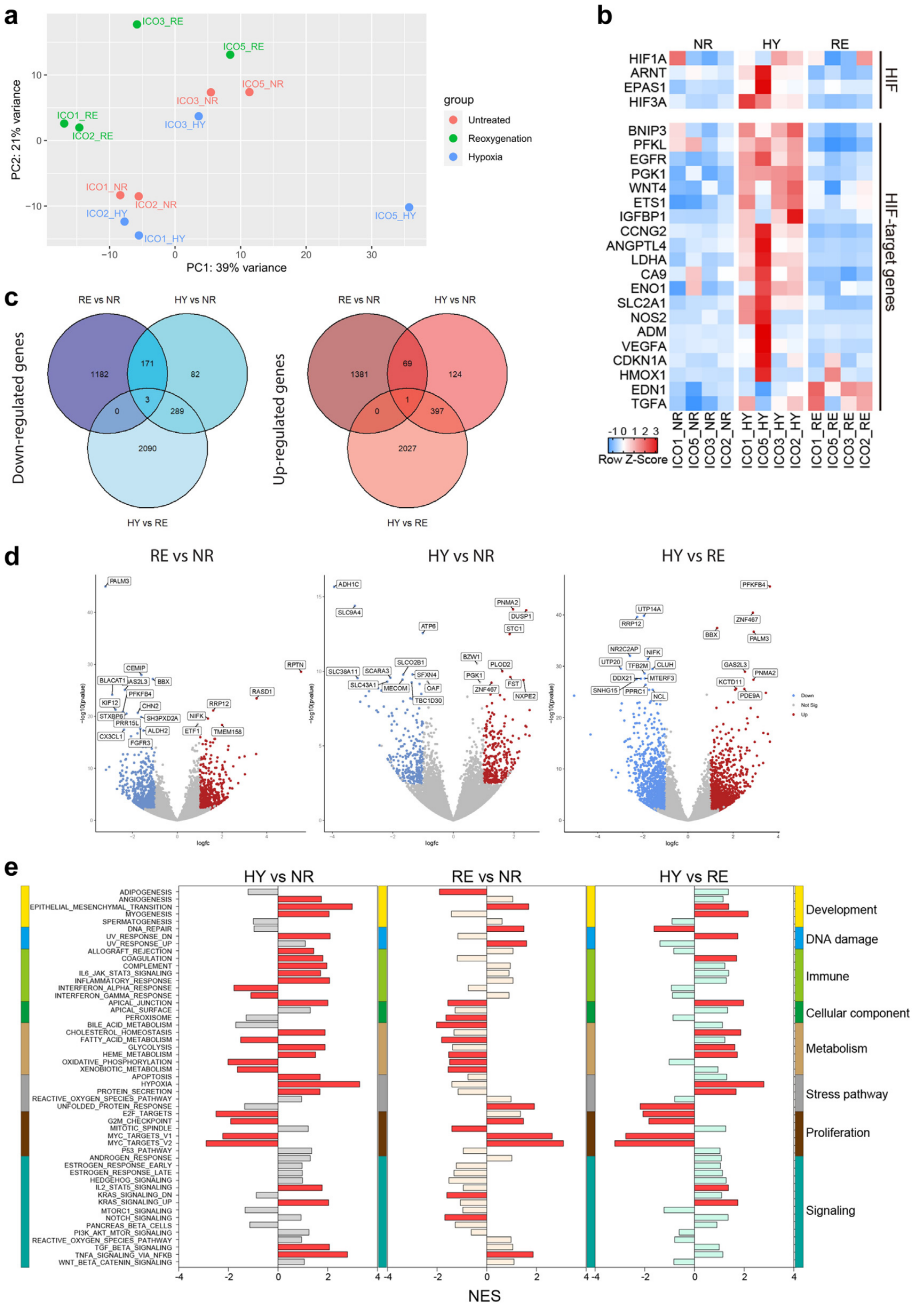
**Transcriptomic analysis of ICOs stressed by H/R injury.** ICOs (n = 4) were stimulated by 72-h hypoxia (HY), followed by 6-h reoxygenation (RE), in which ICOs cultured in normoxia were designated as control (NR). ICOs were lysed immediately after stimulation and bulk RNA sequencing was conducted subsequently. DEGs among different conditions were analyzed based on a threshold of false discovery rate (FDR) t ≤0.05 and fold change ≥2. (a) Principal component analysis was performed based on the DEGs determined by RNA sequence. (b) Heatmap is plotted to show the expression of the HIF family of transcription factors and HIF-1α-regulated genes in ICOs under different conditions. (c) Venn diagram is plotted to show the shared and unique DEGs among ICOs upon H/R stress. (d) Volcano plots showing the results of the RNA sequence. Each dot represents a single gene, with genes that are significantly upregulated shown in red, genes that are significantly downregulated in blue and non-significant genes in gray. (e) Hallmark pathway analysis of RNA-seq experiment. Gene set enrichment analysis for all the 50 hallmark gene sets for 3 different comparisons (HY vs. NR, RE vs. NR, HY vs. RE). The hallmark gene sets were categorized based on the biological processes with unique color labeling, including development, DNA damage, immune, cellular component, metabolism, stress pathway, proliferation, and other signalings. Significantly downregulated (normalized Enrichment Score (NES) < 0) and upregulated (NES > 0) gene sets were labeled in red (nominal p value < 0.05).

To examine the global downstream effect of hypoxia and reoxygenation, the transcriptomes of ICOs cultured in normoxia, hypoxia, and H/R were analyzed by bulk RNA sequencing. A whole-transcriptome comparison between the differently treated ICOs was done using principal component analysis (PCA). ICOs derived from different donors were globally divergent, implying potential individual differences. We found that, though not clearly separated, ICOs with the same stimulation tend to be clustered into 3 groups, suggesting that ICOs share some general responsive features at the transcriptional level (Fig. 3a). Furthermore, expression analysis revealed a global upregulation of the HIF family genes and HIF-1α-regulated genes in ICOs when cultured in hypoxic conditions (Fig. 3b). Interestingly, an inter-donor difference was observed and ICOs derived from donor #5 (ICO5) appear to have the highest expression of hypoxia-related genes upon hypoxia stress. This indicates a donor-specific response to hypoxia in ICOs. Based on a threshold of false discovery rate (FDR) t ≤0.05 and fold change ≥2, a relatively higher number of differentially expressed genes (DEGs) were identified in the reoxygenation phase (2807 vs. 1136) compared with the hypoxia phase. The Venn diagram (Fig. 3c) shows 244 shared DEGs (174 down-regulated and 70 up-regulated). Additionally, 2563 (1182 down-regulated and 1381 up-regulated) and 892 (371 down-regulated and 521 up-regulated) DEGs were unique for the reoxygenation and hypoxia conditions, suggesting that the transcriptional profiles of ICOs during these two phases differ greatly (Fig. 3c). Among the upregulated DEGs expressed in hypoxia and normoxia, ICOs were featured by a hypoxic response, such as *PLOD2*, *DUSP1*, *PGK1*, *PNMA2* and *STC1*, indicating the activation of hypoxia pathways upon hypoxia stress (Fig. 3d).

We further performed gene set enrichment analysis for hallmark gene sets comparing ICOs cultured in hypoxia vs. normoxia, reoxygenation vs. normoxia, and hypoxia vs. reoxygenation (Fig. 3e). For this, 50 hallmark gene sets were grouped into 8 biological processes^27^: development; DNA damage; immune; cellular component; metabolic; stress pathway; proliferation; and other signaling pathways. Compared to normoxia, hypoxia-induced stress resulted in 29 significantly upregulated or downregulated hallmark pathways, while reoxygenation led to significant alterations in 19 pathways. To be more specific, stress pathways were upregulated upon hypoxia cultures, such as hypoxia and apoptosis, while pathways associated with cell proliferation, such as E2F_Targets, G2M_Checkpoint, MYC_Target_V1, and MYC_Target_V2, were downregulated. On the other hand, reoxygenation promoted the downregulation of cellular components and metabolism processes, while pathways associated with cell proliferation were significantly upregulated, suggesting a restored cell proliferating potential. Collectively, hypoxia and reoxygenation stress could result in a distinct transcriptional profile in these two phases.

### Up-regulation of EMT-associated markers during biliary IRI

**Fig. 4:**
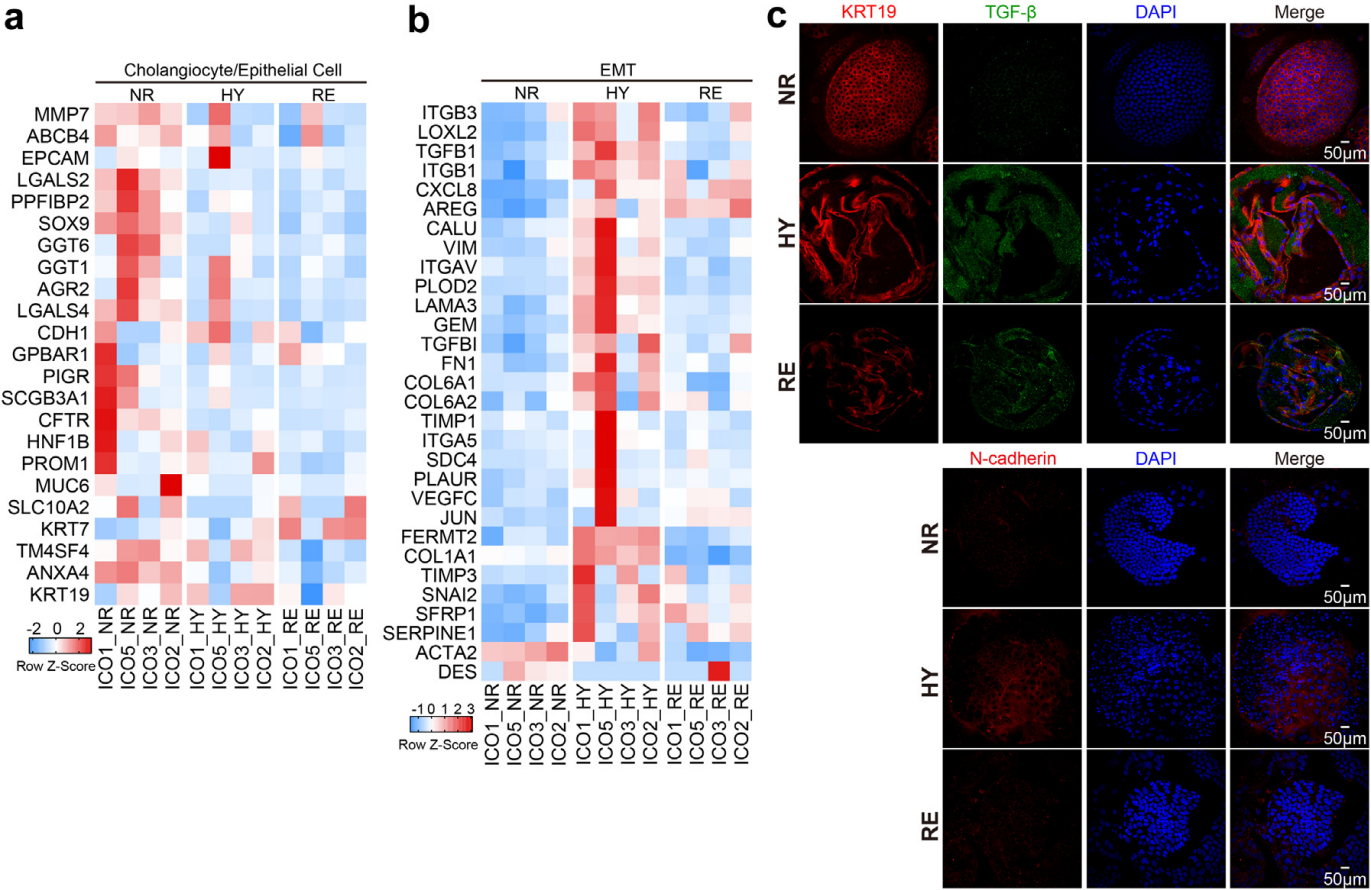
**Upgulaiton of EMT-associated markers in ICOs upon H/R stress.** (a and b) Heatmap is plotted to show the expression of cholangiocyte/epithelial genes and EMT-associated genes in ICOs (n = 4) cultured in normoxia (NR) or 72-h hypoxia (HY) or HY followed by 6-h reoxygenation (RE). (c) Stimulated ICOs were fixed and stained for KRT19, TGF-β, and N-cadherin, along with DAPI staining. Representative fluorescent images are shown.

Biliary EMT defines a process of cellular reprogramming in which biliary epithelial cells acquire morphological and functional phenotypes of mesenchymal cells.^28^ Although still somewhat controversial, EMT of cholangiocytes has been considered an event contributing to biliary fibrosis in diseases such as primary biliary cirrhosis, primary sclerosing cholangitis, and biliary atresia.^14^^,^^29^, ^30^, ^31^, ^32^, ^33^ To investigate the change of expression of EMT-related markers in ICOs stressed by hypoxia and reoxygenation, we further analyzed the RNA sequencing data and found a global downregulation of cholangiocyte-related genes (*MMP7*, *LGALS2*, *SOX9*, *GGT6*, *PPFIBP2*, *CFTR*, *etc.*) and genes expressed in epithelial cells (*ANXA4*, *CD24*, *HNF1B*, *etc.*) after hypoxia treatment (Fig. 4a). Moreover, an obvious upregulation of genes associated with EMT, such as *ITGB3*, *LOXL2*, *VIM*, *TGFB1*, and *PLOD2* was found in ICOs exposed to hypoxia, which was also downregulated drastically after 6-h reoxygenation (Fig. 4b). Notably, the expression of *COL1A1* (collagen I), but not *ACTA2* (alpha-smooth muscle actin), was upregulated clearly after hypoxia stimulation. Immunostaining further confirmed the increased expression of TGF-β and N-cadherin in ICOs as a result of hypoxia treatment (Fig. 4c).

**Fig. 5:**
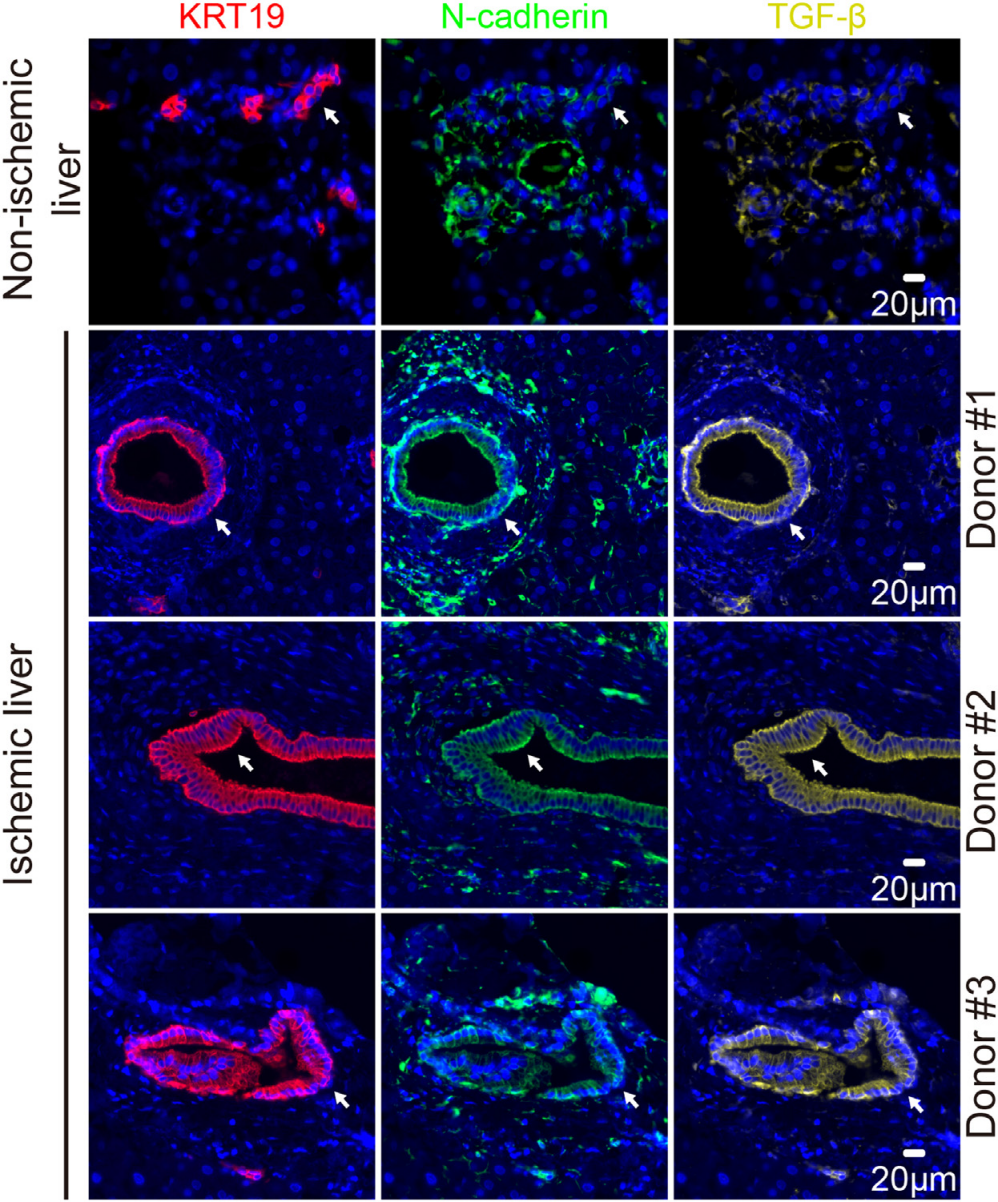
**Increased expression of EMT-associated markers in primary cholangiocytes during clinical liver transplantation IRI.** Liver biopsies from non-ischemia living donor graft (n = 1) of ischemic decreased-donor grafts (n = 3) collected during liver transplantation after reperfusion were fixed and stained using multiplex immunofluorescence for the cholangiocyte-specific marker, KRT19, and EMT-associated markers N-cadherin and TGF-β. White arrows indicate the location of small and large bile ducts.

Recent histological evidence confirmed the activation of TGF-β, a critical player involved in EMT,^34^ in biliary epithelial cells of patients with post-transplant non-anastomotic biliary strictures, and uncovers its potential involvement in biliary fibrosis.^13^ Animal and *in vitro* cell culture models also demonstrate that EMT might be involved in ischemic cholangiopathy.^14^ To investigate the expression of EMT-associated markers during IRI in human liver grafts, we performed multiplex staining on histological sections of deceased donor liver grafts with IRI after transplantation. As a negative control, biopsies from an ischemia-free living donor liver graft were used. The expression of EMT-associated markers, including N-cadherin and TGF-β, was minimally detected in the cells expressing cytokeratin 19 (KRT19), designated as cholangiocytes, in the ischemia-free liver (Fig. 5). On the contrary, we found that part of KRT19 expression overlapped extensively with N-cadherin and TGF-β, suggesting the upregulation of the EMT-related mediators in cholangiocytes. Collectively, these findings demonstrate that the upregulation of EMT-associated markers could be promoted in ICOs upon H/R stress and liver transplant biopsies.

### H/R induces phase-specific cell death patterns in ICOs

**Fig. 6:**
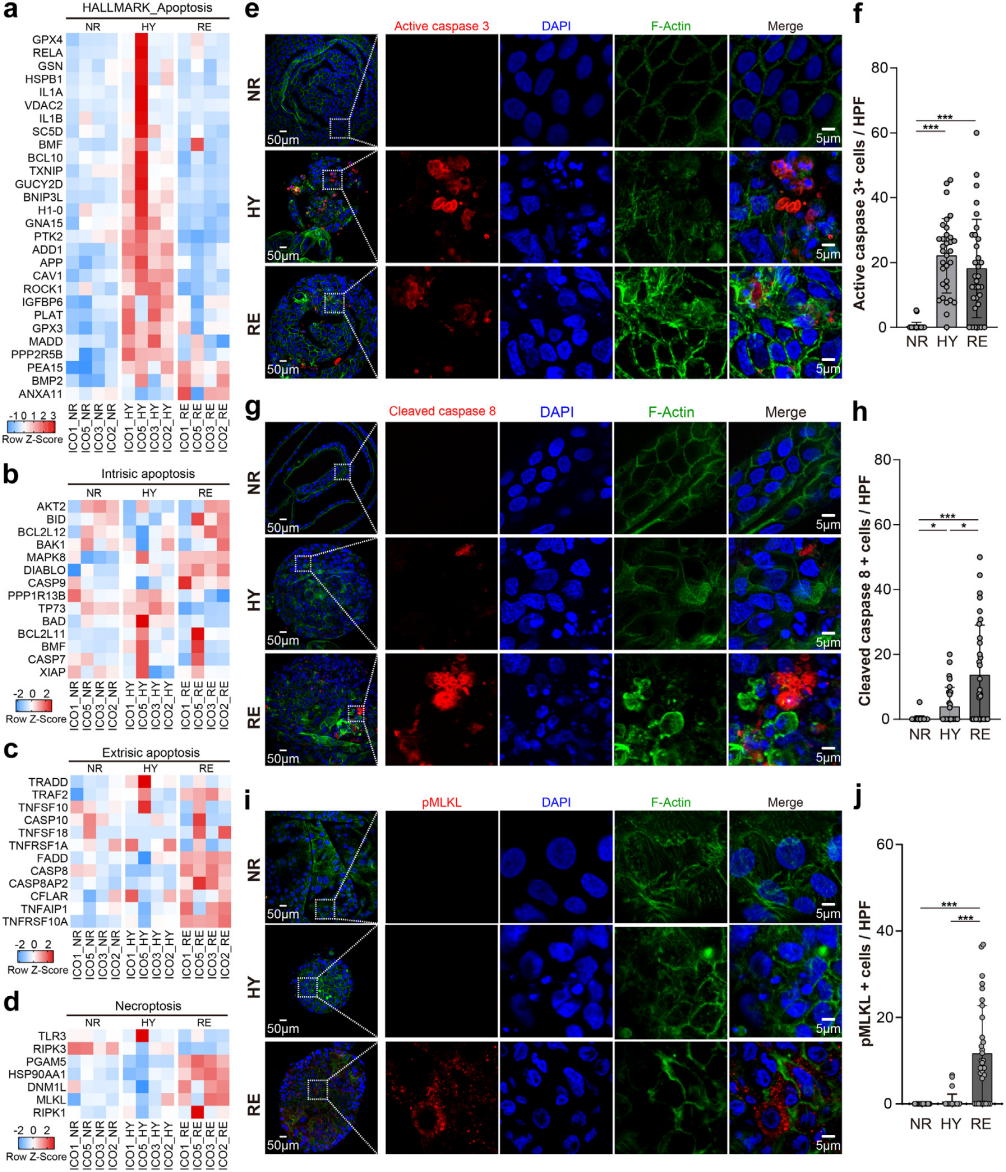
**Programmed cell death induced in ICOs upon H/R stress.** ICOs (n = 4) were stimulated with 72-h hypoxia (HY), followed by 6-h reoxygenation (RE). Based on the RNA-seq experiment, heatmaps are plotted to show the expression of programmed cell death-related genes, including the hallmark apoptosis pathway (a), intrinsic apoptosis (b), extrinsic apoptosis (c), and necroptosis (d), in ICOs upon H/R stress. (e, g and i) Stimulated ICOs (n = 4) were fixed and stained for active caspase 3, cleaved caspase 8, and pMLKL, along with DAPI/F-actin staining. Representative fluorescent images are shown. Enlarged images from the boxed area are shown in the right panels. (f, h and j) Quantification of positive cells in fluorescent images was performed using Image J (at least 8 HPF in each ICOs line and condition). Data are mean ± SD, ∗*p* < 0.05, ∗∗∗*p* < 0.001, Kruskal–Wallis test followed by Dunn's post hoc test (f, h and j).

Hepatic IRI induces different programs of cell death, such as apoptosis, necrosis, autophagy, and necroptosis, depending on the distinct pathogenetic mechanisms during ischemia and reperfusion. This results in biliary epithelial cell loss, which could, in turn, cause post-transplant biliary complications.^35^ Previous clinical and experimental evidence demonstrates that hepatic IRI contributed to apoptosis and necrosis in biliary epithelial cells.^10^, ^11^, ^12^ Our previous study also shows that ICOs could undergo apoptosis or necroptosis, depending on the stimuli.^15^ We reasoned that the distinct phases of IRI may evoke disparate programmed cell death mechanisms, including apoptosis and necroptosis, in ICOs. To this end, we analyzed the transcriptional profile of apoptosis- and necroptosis-associated genes in the stimulated ICOs. We found that the hallmark apoptosis gene sets were upregulated in ICOs grown under hypoxic conditions, and also partially after oxygen reintroduction, suggesting apoptosis was caused by H/R (Fig. 6a). To identify the underlying mechanisms of apoptosis, both intrinsic apoptosis- and extrinsic apoptosis-associated genes were analyzed. The intrinsic apoptosis genes were upregulated in both hypoxia and reoxygenation conditions (Fig. 6b). We observed an obvious upregulation of extrinsic apoptosis-related genes (such as *TRAF2*, *CASP8*, and *TNFRSF10A*) in reoxygenated ICOs, but not in the hypoxia-treated group, indicating that the apoptosis machinery induced by hypoxia and reoxygenation may differ (Fig. 6c). Surprisingly, reoxygenation, rather than hypoxia, also appears to upregulate the necroptosis-associated genes (such as *MLKL*, *PGAM5*, and *DNM1L*) (Fig. 6d).

To support these findings, immunostaining for critical apoptosis and necroptosis markers, including active caspase 3, cleaved caspase 8, and pMLKL, were performed. As shown in Fig. 6e and f, clear activation of caspase 3 was observed in the ICOs treated by either hypoxia (*p* < 0.001, Kruskal–Wallis test followed by Dunn's post hoc test) or subsequent reoxygenation (*p* < 0.001, Kruskal–Wallis test followed by Dunn's post hoc test), suggesting the induction of apoptosis in ICOs. Furthermore, we found extensive expression of cleaved caspase 8 in ICOs stressed by reoxygenation, which was sporadically expressed in cells in the hypoxia condition (mean 13.67 ± 15.38 vs. 2.88 ± 5.95, *p* < 0.05, Kruskal–Wallis test followed by Dunn's post hoc test). This indicates the induction of extrinsic apoptosis in these cells, particularly at the reoxygenation phase (Fig. 6g and h). Moreover, the induction of necroptosis was further confirmed by the apparent pMLKL activation in ICOs under reoxygenation, but not hypoxia, culture conditions (*p* < 0.001, Kruskal–Wallis test followed by Dunn's post hoc test), which implies that necroptosis occurs exclusively during reoxygenation (Fig. 6i and j). To summarize, these findings confirm that apoptosis and necroptosis could be induced in ICOs grown under IRI-mimicking cultures in a phase-specific manner.

### ICOs under H/R culture as a model for identification of cholangio-protective agents

**Fig. 7:**
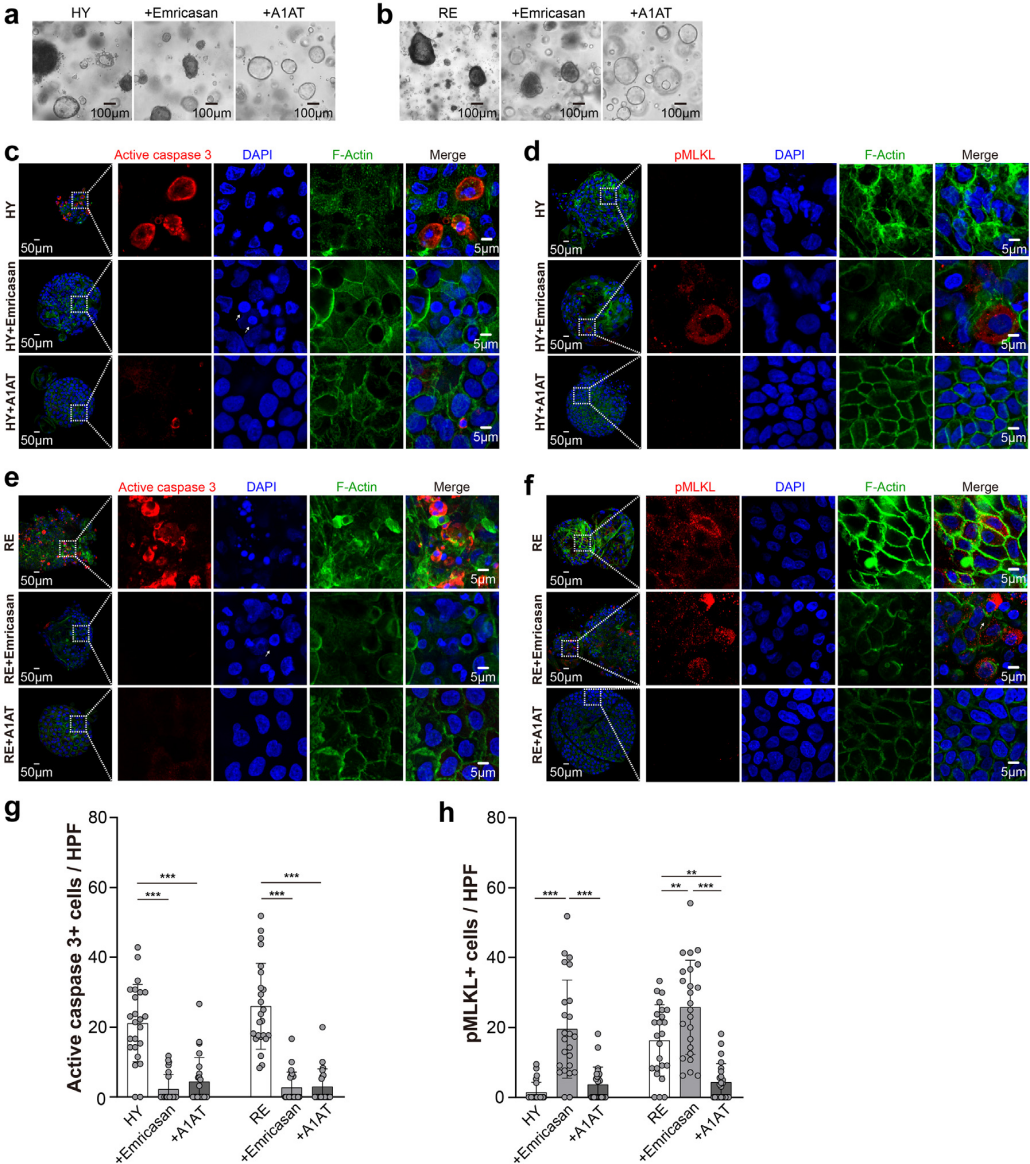
**Drug evaluation and identification using the H/R model on ICOs.** ICOs (n = 3) were stimulated with 72-h hypoxia (HY), followed by 6-h reoxygenation (RE), in the presence or absence of emricasan (20 μM) or A1AT (20 mg/mL). (a and b) Representative bright-field images of the treated ICOs are shown. (c–f) Stimulated ICOs (n = 3) were fixed and stained for active caspase 3 and pMLKL, along with DAPI/F-actin staining. Representative fluorescent images are shown. Enlarged images from the boxed area are shown in the right panels. (g and h) Quantification of positive cells in fluorescent images was performed using Image J (at least 8 HPF in each ICOs line and condition). Data are mean ± SD, ∗∗*p* < 0.01, ∗∗∗*p* < 0.001, Kruskal–Wallis test followed by Dunn's post hoc test (g and h).

Next, we deployed the ICOs under H/R culture as a preclinical drug screening tool to identify cholangio-protective agents. Emricasan (IDN-6556) is a potent oral caspase inhibitor that is known to improve liver function in patients with chronic liver diseases.^36^^,^^37^ However, recent randomized trials show that emricasan failed to benefit patients with non-alcoholic steatohepatitis (NASH)-related cirrhosis and may inversely promote alternative cell death and worsen hepatic fibrosis and ballooning.^38^ To evaluate the inhibitory effect of emricasan on H/R-induced damage, the ICOs were co-incubated with emricasan throughout the hypoxia and reoxygenation cultures. Interestingly, the treatment with emricasan failed to rescue the ICOs under either hypoxia or reoxygenation conditions, in which the ICOs displayed gray and intact lumens and small rounded and pyknotic nuclei, resembling a necroptosis-like phenotype^15^ (Fig. 7a and b). We found that emricasan efficiently suppressed the caspase 3 activity in ICOs stressed by hypoxia (*p* < 0.001, Kruskal–Wallis test followed by Dunn's post hoc test) and reoxygenation (*p* < 0.001, Kruskal–Wallis test followed by Dunn's post hoc test) (Fig. 7c, e, and g). After supplementing the cultures with emricasan, substantial expression of pMLKL was observed in ICOs cultured in hypoxic conditions, suggesting that caspase inhibition invoked necroptotic cell death (Fig. 7d and h). Similarly, an increase of pMLKL^+^ cells was observed in the ICOs stressed by reoxygenation, and that was exposed with emricasan (25.78 *± 13.46*% vs. 16.30 *± 10.23*, *p* < 0.01, Kruskal–Wallis test followed by Dunn's post hoc test) (Fig. 7f and h).

Alpha1-antitrypsin (A1AT) is an acute phase reactant serine protease inhibitor for the treatment of patients with A1AT deficiency, which also exerts cytoprotective capacity against IRI in various organs including lung,^39^ pancreas,^40^ heart,^41^ and kidney.^42^ We investigated whether exposure to A1AT could alleviate H/R-induced injury in ICOs. We found that A1AT restored the epithelial structure of ICOs cultured in hypoxic conditions and during reoxygenation (Fig. 7a and b), suggesting a potential cytoprotective effect. As shown in Fig. 7c, e, and g, the treatment of A1AT significantly suppressed the caspase 3 activation in ICOs induced by either hypoxia (*p* < 0.001, Kruskal–Wallis test followed by Dunn's post hoc test) or reoxygenation (*p* < 0.001, Kruskal–Wallis test followed by Dunn's post hoc test). Co-incubation of ICOs with A1AT during hypoxia didn't result in MLKL phosphorylation (Fig. 7d and h). Of note, the activation of pMLKL during reoxygenation was also obviously retained by A1AT (*p* < 0.01, Kruskal–Wallis test followed by Dunn's post hoc test) (Fig. 7f and h). Taken together, these data demonstrate that caspase inhibition promotes necroptosis during episodes of hypoxia and reoxygenation, while A1AT exerts the protective property against hypoxia- and reoxygenation-induced injury.

## Discussion

IRI is a challenging issue in clinical practice, especially in liver transplantation, in which the detailed pathogenic mechanism in the bile duct remains unclear. In this study, we demonstrate the use of ICOs to model ischemic biliary injury *in vitro*. Transcriptome analysis and molecular readout provide new insights into the response of biliary epithelial cells to H/R and allows for the identification of pharmacological interventions.

We previously described that cholangiopathy-associated stimuli-induced damage in ICOs provides a set of methodologies for evaluating and validating biliary epithelial injury in three-dimensional culture.^15^ In this study, we found a similar morphological pattern in ICOs stressed by H/R. Generally, the size of ICOs decreased noticeably under hypoxia compared to normoxia conditions, possibly resulting from the reduced proliferation or initiated cell death programs. The morphological features of apoptotic and necroptotic ICOs are consistent with what we described before.^15^ The disintegration of the actin cytoskeleton indicated impaired epithelial integrity under both hypoxia and reoxygenation stress. The nuclei in ICOs under H/R conditions showed an irregular shape, while most nuclei in TNF-α, human bile, or ethanol metabolites treated ICOs display an oval shape with fragments.^15^

The transcriptome analysis revealed a landscape of pathophysiological genetic changes in ICOs responding to H/R injury. The HIF family genes, as well as HIF-target genes, show a general upregulation after hypoxia stimulation but could be recovered by reintroduction to normoxia, suggesting a typical response to hypoxia. The majority of previous clinical and experimental studies investigated ischemic cholangiopathy in a general way, in which the distinct biliary injury mechanisms during IRI have rarely been studied. Analysis of gene sets of particular pathways further demonstrates the phase-dependent transcriptional change of ICOs upon H/R insults. For instance, hypoxia alone could activate the EMT- and apoptosis-associated pathways and suppress the cell proliferation process in ICOs, which could be restored in the reoxygenation phase. On the contrary, the death receptor-mediated programmed cell death, such as extrinsic apoptosis and necroptosis, appear to be predominant in the reoxygenation phase. Ferreira-Gonzalez et al.^43^ demonstrated cholangiocyte senescence as a detrimental mechanism in the development of biliary injury. Interestingly, we also found a general upregulation of senescence-associated genes in ICOs after reoxygenation rather than the hypoxia phase (data not shown), possibly linking to the biliary injury mechanism during reoxygenation. Of note, the phase-specific damage patterns have been deeply studied in hepatocellular damage during IRI,^43^ while few were reported in the biliary injury. Our organoid-based study provides a better understanding of the transcriptional changes of biliary epithelial cells upon H/R stress and may facilitate the identification of ischemia- or reperfusion-targeted treatments. Besides, the bulk RNA sequencing used in our study provides a general transcriptional profile of stimulated ICOs. However, based on our results, multiple pathways were activated in the ICOs in the same or different cells and their interaction is unclear. Techniques such as single-cell RNA sequencing are worth to be applied in the future.

Although the induction of EMT in cholangiopathies is still controversial, EMT in cholangiocytes represents a critical player contributing to biliary fibrosis in patients with primary biliary cirrhosis, primary sclerosing cholangitis, and biliary atresia.^29^, ^30^, ^31^, ^32^, ^33^^,^^44^ A few studies demonstrate that EMT is also involved in ischemic cholangiopathy.^14^ Based on histological assessment of clinical liver graft biopsies collected during liver transplantation, we confirmed the expression of EMT-associated markers in biliary cells in deceased donor liver transplantation, but not non-ischemic, biopsies. The EMT-associated genes were mostly activated during hypoxia and could be suppressed after reoxygenation in ICOs, suggesting its reversible potential. Whether the upregulation of EMT-associated markers was induced in the ICOs by hypoxia directly^45^ or by TGF-β in an autocrine or paracrine manner^34^ is yet unknown. The exact effect of EMT induced by acute IRI still remains unclear. We speculate that the increased expression of EMT-related markers in ICOs might be an adaptive response in response to hypoxia insults,^46^ which could be reverted after oxygen reintroduction. This process might also be associated with biliary regeneration and post-transplant fibrosis, which is worth to be further investigated. Of note, according to the latest guidelines and definitions regarding the investigation of EMT, the activation of EMT cannot be simply identified based on the upregulation of a few molecular markers.^47^ Thus, whether the EMT was activated or not during biliary IRI remains to be further clarified. Moreover, the ICOs culture partially resembles mature primary cholangiocytes *in vitro* but also represents cholangiocytes with higher proliferating activity and stemness due to the canonical Wnt-stimulating culture condition,^25^^,^^26^ which may provide a new platform to investigate the biliary regeneration under IRI.

The balance between biliary regeneration and cell death is critical for homeostasis in the biliary system. The cellular and mitochondrial perturbations during cold preservation, including depletion of ATP and cell swelling, contribute to oncotic necrosis in both hepatocytes and cholangiocytes.^6^ However, cholangiocytes are more susceptible to IRI compared to hepatocytes, particularly in the reoxygenation phase, due to slower ATP regeneration, more oxidative products, and impaired antioxidant potential.^5^ Except for apoptosis and necrosis, we previously described that cholangiocytes could undergo necroptosis, an emerging known type of regulated necrosis, upon acute injury, simultaneously evoked with apoptosis in some cases.^15^ In this study, based on ICOs culture, we further uncovered the phase-dependent cell death machinery in cholangiocytes during IRI. The intrinsic apoptosis represents the predominant programmed cell death during hypoxia culture. Differently, both extrinsic apoptosis and necroptosis could be induced after reoxygenation. Of note, our knowledge of the regulated necrosis network, such as necroptosis, ferroptosis, and pyroptosis has been expanded in the last decades. With an eye on apoptosis and necroptosis in our study, we cannot exclude the involvement of other regulated necrosis modalities to promote ischemia cholangiopathy. Future investigation focusing on the role of multiple programmed cell death in ischemic cholangiopathy should be performed.

The identification of precise cell death type points out ICOs as a promising drug evaluation and identification tool. For instance, caspase inhibiting agents, such as emricasan, have been widely examined in clinical trials but show conflicting results, and do not show patient benefits in fatty liver disease.^38^ Our previous study demonstrates that a supplement of caspase inhibition could not rescue extrinsic apoptosis but switch it into necroptosis, which holds stronger immunogenic nature than apoptosis.^15^ This might be an explanation for the worsened fibrosis and hepatocyte ballooning in the emricasan trial.^38^ We here reported that emricasan enhances cell death by promoting necroptosis at both hypoxia and reoxygenation phases, raising the concern about the application of caspase inhibitors in liver transplantation. Inversely, administration of A1AT, an acute phase reactant serine protease inhibitor, reduced apoptosis and necroptosis at both hypoxia and reoxygenation phases. Given that A1AT is mainly synthesized in hepatocytes, it would be interesting to further investigate the interaction between hepatocytes and cholangiocytes during hepatic IRI.

During liver transplantation, the biliary cells respond to IRI in a highly dynamic way. This greatly limits current clinical histological studies because of the difficulty to collect biopsies at appropriate time points. Based on cholangiocyte organoids, our IRI model provides a useful *in-vitro* tool to monitor the response of cholangiocytes to IRI in a real-time and precise manner. The biliary IRI model can serve the clinic from the perspective of the pathogenesis of ischemic biliary injury. Considering the lack of an effective animal model for ischemic cholangiopathy, we assume that the combination of our organoid-based biliary IRI model (drug identification) and *ex vivo* machine perfusion (drug validation) will be a promising strategy to develop sufficient pharmacological interventions for the clinical management of ischemic cholangiopathy. For instance, the supplement of A1AT, identified by our IRI model, during *ex vivo* machine perfusion represents a promising next step to prevent ischemic biliary injury during liver transplantation. However, it must be admitted that the ischemic damage in cholangiocyte organoids cannot fully recapitulate the ischemic cholangiopathy pathogenesis in patients because of the lack of other liver cells, such as immune cells and mesenchymal cells. The co-culture system might be a solution to optimize the biliary IRI model in the future.

In conclusion, our study shows that human cholangiocyte organoids represent a good model for ischemia and reperfusion injury of bile ducts *in vitro*. The results described here may add to, so far limited, the knowledge of the ischemic biliary injury. The model described provides a useful pre-clinical platform to investigate the pathogenesis of ischemic cholangiopathies and the assessment of drugs to prevent this.

## Contributors

Conceptualization: L.J.W.v.d.L., M.M.A.V., S.S.; Methodology: S.S., H.P.R., T.P.P.v.d.B., M.J.C.B., M.U.B., J.d.J., S.O.D., A.A.F.d.V., H.R.d.J., M.M.A.V., L.J.W.v.d.L. Investigation: S.S., M.M.A.V., L.J.W.v.d.L. Visualization: S.S., H.P.R., T.P.P.v.d.B. Supervision: M.M.A.V., L.J.W.v.d.L. Writing—original draft: S.S. Writing—review & editing: S.S., H.P.R., T.P.P.v.d.B., M.J.C.B., M.M.A.V., L.J.W.v.d.L. All authors read and approved the final version of the manuscript for publication. More than one author, including L.J.W.v.d.L., M.M.A.V., S.S., H.P.R., T.P.P.v.d.B., has directly accessed and verified the underlying data reported in the manuscript. L.J.W.v.d.L. and M.M.A.V. were responsible for the decision to submit the manuscript.

## Data sharing statement

The datasets generated during the current study will be available from the corresponding author upon reasonable request.

## Declaration of interests

The authors declared there are no conflicts of interest.
