# Supplementary material for: Modeling bile duct ischemia and reoxygenation injury in human cholangiocyte organoids for screening of novel cholangio-protective agents
Source: eBioMedicine. 2023 Jan 4;88:104431. doi: 10.1016/j.ebiom.2022.104431 (PMC9826934; doi:10.1016/j.ebiom.2022.104431)
Supplement: Supplemental Tables S1–S3 [file mmc1.docx]

**Supplementary tables:**

**Table S1** The main demographic and clinical characteristics of donors for immunohistochemistry analysis.

###

|  | Donor liver 1 | Donor liver 2 | Donor liver 3 |
| --- | --- | --- | --- |
| Sex (F/M) | F | F | F |
| Age (years) | 64 | 77 | 43 |
| BMI (kg/m^2^) | 21.48 | 24.5 | 22.4 |
| AST(U/L) | 52 | 39 | 81 |
| ALT (U/L) | 22 | 49 | 55 |
| GGT (U/L) | 106 | 59 | 50 |
| Total bilirubin(mg/dL) | 4 | 6 | 8 |
| HBV (Y/N) | N | N | N |
| HCV (Y/N) | N | N | N |

**Table S2** List of media compositions used in cholangiocyte organoid culture in this study.

| Components | Concentration | Source |
| --- | --- | --- |
| N2 | 1% | Gibco |
| B27 | 1% | Gibco |
| N-Acetylcysteine | 1.25 mM | Sigma-Aldrich |
| gastrin | 10 nM | Sigma-Aldrich |
| EGF | 50 ng/ml | Peprotech |
| FGF10 | 100 ng/ml | Peprotech |
| HGF | 25 ng/ml | Peprotech |
| R-spondin | 10% | Conditioned medium |
| nicotinamide | 10 nM | Sigma-Aldrich |
| A83.01 | 5 µM | Tocris |
| forskolin | 10 µM | Tocris |
| Noggin | 25 ng/ml | Conditioned medium |
| Wnt | 30% | Conditioned medium |
| Y27632 | 10 µM | Sigma-Aldrich |
| hES cell cloning recovery supplement | 2 µM | Stemgent |

**Table S3** List of antibodies used in this study.

| Antibody | Reactivity | Application | Species | | Source | Dilution |
| --- | --- | --- | --- | --- | --- | --- |
| pMLKL | Human | IF | Rabbit | Polyclonal | RRID:AB_2817106 | 1:100 |
| Active Caspase 3 | Human/ Mouse | IF | Rabbit | Polyclonal | RRID:AB_2243952 | 1:40 |
| Cleaved caspase 8 | Human/ Mouse | IF | Rabbit | Monoclonal | RRID:AB_561381 | 1：250 |
| KRT19 | Human | IF | Mouse | Monoclonal | RRID:AB_1158242 | 1.4 µg/ml |
| Ki67 | Human | IF | Rabbit | Monoclonal | RRID:AB_2631262 | 2 μg/mL |
| TGF-β | Human | IF | Rabbit | Polyclonal | RRID:AB_2202305 | 1:100 |
| N-cadherin | Human | IF | Mouse | Monoclonal | RRID:AB_2077542 | 1.4 µg/ml |
